# Phosphorylation-Independent Regulation of Atf1-Promoted Meiotic Recombination by Stress-Activated, p38 Kinase Spc1 of Fission Yeast

**DOI:** 10.1371/journal.pone.0005533

**Published:** 2009-05-14

**Authors:** Jun Gao, Mari K. Davidson, Wayne P. Wahls

**Affiliations:** Department of Biochemistry and Molecular Biology, University of Arkansas for Medical Sciences, Little Rock, Arkansas, United States of America; University of Missouri-Kansas City, United States of America

## Abstract

**Background:**

Stress-activated protein kinases regulate multiple cellular responses to a wide variety of intracellular and extracellular conditions. The conserved, multifunctional, ATF/CREB protein Atf1 (Mts1, Gad7) of fission yeast binds to *CRE*-like (*M26*) DNA sites. Atf1 is phosphorylated by the conserved, p38-family kinase Spc1 (Sty1, Phh1) and is required for many Spc1-dependent stress responses, efficient sexual differentiation, and activation of Rec12 (Spo11)-dependent meiotic recombination hotspots like *ade6-M26*.

**Methodology/Principal Findings:**

We sought to define mechanisms by which Spc1 regulates Atf1 function at the *ade6-M26* hotspot. The Spc1 kinase was essential for hotspot activity, but dispensable for basal recombination. Unexpectedly, a protein lacking all eleven MAPK phospho-acceptor sites and detectable phosphorylation (Atf1-11M) was fully proficient for hotspot recombination. Furthermore, tethering of Atf1 to *ade6* in the chromosome by a heterologous DNA binding domain bypassed the requirement for Spc1 in promoting recombination.

**Conclusions/Significance:**

The Spc1 protein kinase regulates the pathway of Atf1-promoted recombination at or before the point where Atf1 binds to chromosomes, and this pathway regulation is independent of the phosphorylation status of Atf1. Since basal recombination is Spc1-independent, the principal function of the Spc1 kinase in meiotic recombination is to correctly position Atf1-promoted recombination at hotspots along chromosomes. We also propose new hypotheses on regulatory mechanisms for shared (*e.g.*, DNA binding) and distinct (*e.g.*, osmoregulatory *vs.* recombinogenic) activities of multifunctional, stress-activated protein Atf1.

## Introduction

Homologous recombination is induced to high levels in meiosis and produces crossover recombination structures that help to align paired homologous chromosomes on the metaphase plate in preparation for the first (reductional) meiotic division [1]. Meiotic recombination is not distributed randomly, but is clustered preferentially at hotspots [2]–[4]. The aberrant segregation of chromosomes in meiosis is the leading cause of spontaneous pregnancy loss, congenital birth defects, and mental retardation in humans; and these errors are almost always associated with defects in the positioning or frequency of recombination [5]. Thus in addition to being a topic of fundamental biological interest, the mechanisms for the correct positioning of meiotic recombination are of biomedical importance.

**Figure 1:**
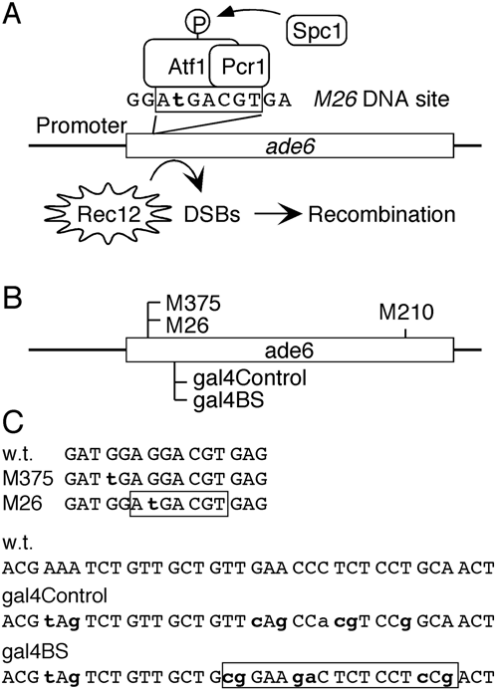
Characteristics of the experimental system. (A) Relevant features of meiotic recombination hotspot *ade6-M26*. The Spc1 kinase-regulated, Atf1-Pcr1-*M26* protein-DNA complex promotes the initiation of meiotic recombination catalyzed by meiotic recombination protein Rec12 (Spo11). (B) Relative positions of *ade6* alleles used in this study. (C) DNA sequences of *ade6* alleles. Individual mutations are depicted by bold lower case. Each mutant allele encodes a translational stop codon (5′-UGA-3′ or 5′-UAG-3′). The *ade6-M26* allele creates a DNA binding site for Atf1-Pcr1 heterodimer (box), its matching negative control allele *ade6-M375* does not. The *ade6-gal4BS* allele creates a DNA binding site for Gal4 protein (box), its matching negative control allele *ade6-gal4Control* does not.

The *ade6-M26* recombination hotspot of fission yeast is the most extensively characterized and best understood meiotic hotspot in any eukaryote (**Figure 1**) (see ref. [6] for recent review). The *ade6-M26* allele promotes specifically meiotic (but not mitotic) recombination [7]. This allele has a single base pair substitution [8], [9] which created a seven base pair, *CRE*-like DNA site (*M26*, 5′-ATGACGT-3′) [10] that is bound by Atf1-Pcr1 (Mts1-Mts2) heterodimer *in vitro* [11] and *in vivo* [12]. The Atf1-Pcr1-*M26* protein-DNA complex is essential for hotspot activity at *ade6-M26* [13]. However, the homologous recombination-activation (HRA) domain of Atf1-Pcr1 heterodimer resides exclusively in Atf1 [14]. Pcr1 helps Atf1 to confer DNA binding site specificity of the heterodimer *in vitro* [11] and *in vivo* [12], [14]. (The DNA binding domain of each protomer binds to one half-site of the whole DNA site that is recognized by the heterodimer.) Engineered and naturally occurring *M26* or *M26*-like DNA sites elsewhere in the fission yeast genome are hotspots and, to the extent tested, their mechanism of function recapitulates that at *ade6-M26* [15], [16]. It has been calculated that such DNA sites could regulate about half of meiotic recombination in the genome [12], [16]. The Atf1-Pcr1-*M26* protein-DNA complex promotes, locally and in *cis*, the initiation of recombination from dsDNA breaks catalyzed by Rec12 (Spo11) [17], which is a broadly conserved protein of the basal meiotic recombination machinery [18]–[21]. The recombination-activation domain of Atf1 sufficient to promote recombination is well conserved in other eukaryotes [14], although its functionality in those organisms has not been reported. And to the extent tested, the general mechanisms for Atf1-promoted recombination seem to be employed in other organisms (*e.g.*, [22], [23]). Therefore, the *ade6-M26* hotspot provides a useful paradigm for how meiotic recombination is distributed preferentially to hotspot sites on chromosomes.

The Atf1 (Mts1, Gad7) and Pcr1 (Mts2) proteins are members of the ATF/CREB family of transcription factors [11], [13], [24]–[28]. Atf1 is phosphorylated by the multifunctional, stress-activated, p38-family protein kinase Spc1 (Sty1, Phh1) [25], [28]. Furthermore, the Spc1 kinase and Atf1 protein are mutually required for a broad range of diverse stress responses, including the induction of sexual differentiation (and hotspot recombination) when nutrients are limiting (*e.g.*, [12]–[14], [25]–[36]). Protein kinases that phosphorylate transcription factors can exert their regulation by altering sub-cellular localization, by affecting protein conformation or stability, by controlling protein-protein interactions, by modulating protein-DNA interactions, and by delivering the kinases to additional target proteins at transcription factor binding sites. Each of these mechanisms has been hypothesized to regulate the mutually interdependent functions of Spc1 and Atf1 [12], [25], [28], [33], [35], [37]–[41]. We therefore sought to test these hypotheses and to further define the mechanisms by which Spc1 kinase regulates Atf1-promoted meiotic recombination at *ade6-M26*.

## Results

Binding of Atf1-Pcr1 heterodimer to a *CRE*-like DNA site (*M26*) promotes local meiotic recombination, so to determine whether the hotspot is active we measured the frequency of meiotic recombination between two sets of *ade6* alleles (**Figures 1** **,** **2**). The *ade6-M26* allele contains a DNA binding site for Atf1-Pcr1 heterodimer, whereas the *ade6-M375* and *ade6-M210* alleles do not. Crosses between strains harboring the *ade6-M375* and *ade6-M210* alleles revealed the basal (control) recombination levels, while crosses between strains harboring the *ade6-M26* and *ade6-M210* alleles revealed the *M26* DNA site-dependent (hotspot) recombination levels [7], [13]. An elevated ratio of recombinants (*M26∶M375*) demonstrates that the hotspot is active.

**Figure 2:**
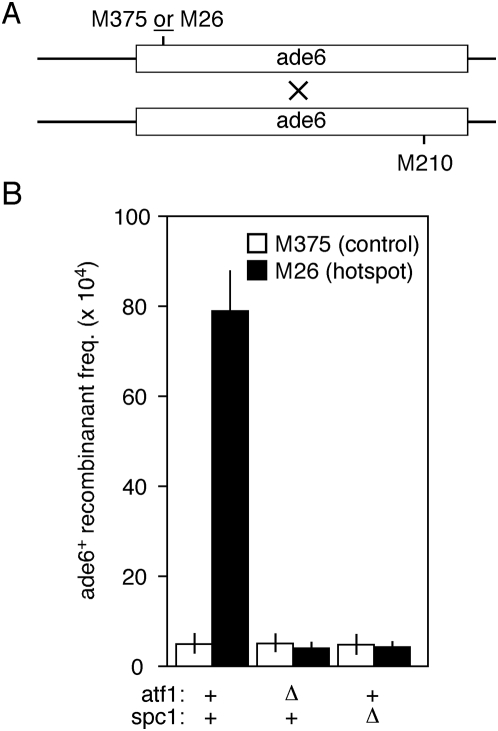
Effects of the *M26* DNA site, Spc1 kinase, and Atf1 protein upon meiotic recombination at *ade6*. (A) Recombination assay. Haploid strains harboring the indicated *ade6* alleles were crossed and, following meiosis, the haploid meiotic products were genotyped to determine the frequencies of *ade6^+^* recombinants. (B) Recombinant frequencies. Cells of the indicated genotypes were tested for their proficiencies of recombination involving hotspot (*M26*) and control (*M375*) alleles of *ade6*. For this and subsequent figures, data are mean±standard deviation from three or more independent experiments.

### The Spc1 kinase, Atf1 protein, and *M26* DNA site function synergistically to promote meiotic recombination

As important controls for the *cis*- and *trans*-acting factors used in this study, we re-examined the roles of Atf1, Spc1, and *M26* in promoting recombination (**Figure 2**). In cells expressing wild-type Atf1 protein, the frequency of *ade6^+^* recombinants from crosses harboring the hotspot (*M26*) allele was significantly higher than that from crosses harboring the control (*M375*) allele, which demonstrates that the hotspot is active. In the *atf1Δ* (null) mutants, the frequency of recombinants from control (*M375*) crosses was similar to that of cells expressing wild-type Atf1 protein, demonstrating that Atf1 is not required for basal recombination. However, the *M26* DNA site-dependent increase in recombination was abolished, confirming that Atf1 is strictly required to activate the *ade6-M26* meiotic recombination hotspot [13]. In the *spc1Δ* mutants, recombination at *M26* was also decreased to basal (*M375*) levels, which confirms that the Spc1 protein kinase is also essential for hotspot activity [12].

### Atf1 is rate-limiting for hotspot recombination

**Figure 3:**
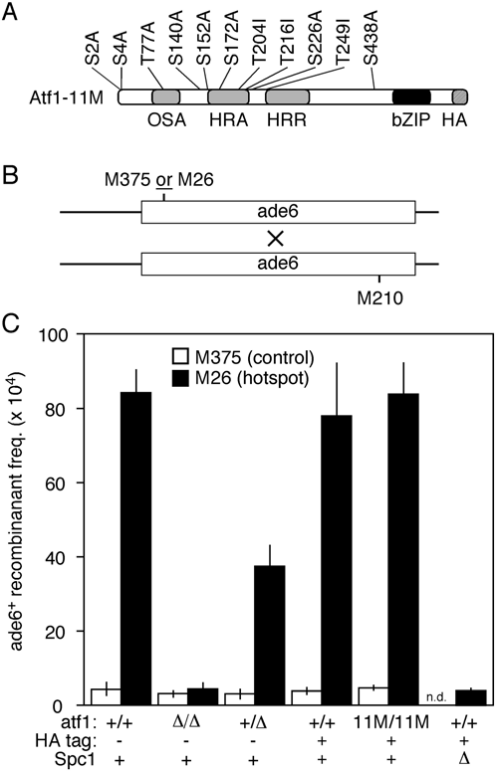
Effects of *atf1* gene dosage and phosphorylation of Atf1 protein upon hotspot meiotic recombination. (A) Diagram of non-phosphorylatable Atf1-11M protein showing the positions of amino acid substitutions and the HA epitope tag [40], relative to the positions of experimentally defined, functionally important regions [14]. These are osmotic stress activation (OSA), homologous recombination activation (HRA), homologous recombination repression (HRR), and basic leucine zipper (bZIP). An HA epitope-tagged version of wild-type (phosphorylatable) Atf1 protein was used as a matching control. (B) Recombination assay. Strains harboring the indicated *ade6* alleles were crossed and the meiotic products were genotyped to determine the frequencies of *ade6^+^* recombinants. (C) Recombinant frequencies. Cells of the indicated genotypes were tested for their proficiencies of recombination involving hotspot (*M26*) and control (*M375*) alleles of *ade6*. *n.d.*, not determined.

There are approximately 200 DNA binding-proficient Atf1 protein molecules per cell [11] and there are about 300 to 800 DNA binding sites for Atf1 per genome (depending upon whether one uses a strict or relaxed consensus for the *CRE*-like, *M26* DNA site) [13], [16]. We therefore determined the effects of *atf1^+^* gene dosage upon hotspot recombination at *ade6-M26* (**Figure 3**). In crosses between two *atf1^+^* parents (homozygous *atf1^+^/atf1^+^*), the recombinant frequency for hotspot allele *ade6-M26* was significantly higher than that for control allele *ade6-M375*. In crosses homozygous for *atf1Δ*, the recombinant frequency for *ade6-M26* was decreased to basal (*ade6-M375*) levels. And in the heterozygous crosses between *atf1^+^* and *atf1Δ* cells, the frequency of recombinants for hotspot allele *ade6-M26* was intermediate between those of homozygous wild-type cells and homozygous null mutants. This phenotype was observed regardless of which *ade6* strain carried the *atf1Δ* allele (data not shown). It can therefore be ascribed to haploinsufficiency in the zygotic diploid meiosis, rather than to an epigenetic effect upon the hotspot allele. In contrast, the frequency of recombinants for control allele *ade6-M375* was not affected significantly by *atf1^+^* dosage (**Figure 3**). In other words, the effects upon *M26*-dependent recombinant frequencies are not due to general defects in meiotic proficiency or basal recombination, but are specific to the hotspot. Therefore the haploinsufficiency indicates that Atf1 is rate-limiting for hotspot recombination, which is consistent with the Atf1 protein∶DNA binding site ratio within cells (above). More to the point, these findings indicate that mutations which have even a minor deleterious effect on the recombination-promoting activity of Atf1 should be readily detectable.

### Phosphorylation of Atf1 at MAPK phospho-acceptor sites is dispensable for hotspot activity

Atf1 is phosphorylated *in vivo* in a Spc1 kinase-dependent manner and Spc1 directly phosphorylates Atf1 *in vitro* [25], [28]. In addition, the Spc1 protein kinase is required for all known Atf1-dependent functions, including hotspot recombination (*e.g.*, [12], [25], [28], [35]). Together, such findings support a general view in which the phosphorylation of Atf1 by Spc1 is regulatory and probably required for Atf1-dependent functions. We therefore examined how the phosphorylation of Atf1 by the Spc1 kinase affects Atf1-Pcr1-*M26* complex-promoted meiotic recombination.

We first determined the proficiencies of recombination for *ade6* alleles in strains with a wild type version of Atf1 protein that harbors two HA epitopes and six histidine residues at its C-terminus (Atf1-HA) (**Figure 3**). The recombinant frequencies for hotspot (*M26*) and control (*M375*) alleles of *ade6* were essentially identical to those of cells expressing wild type, untagged Atf1 protein. We concluded that the epitope-tagged Atf1 protein is fully functional for promoting *M26* DNA site-dependent, hotspot recombination at *ade6-M26*.

We then determined the frequencies of recombination for *ade6* alleles in strains expressing a phosphorylation-deficient version of Atf1 (Atf1-11M-HA) [40]. In this protein all eleven potential MAPK phospho-acceptor sites, which are a serine or threonine residue immediately followed by a proline, have been replaced with alanine or isoleucine (**Figure 3A**). The Atf1-11M-HA protein is not phosphorylated by Spc1 or by any other protein kinase, as judged by two-dimensional mapping of phosphorylation events [40]. (It remains formally possible that this phosphorylation-deficient Atf1 protein is phosphorylated at non-canonical phospho-acceptor sites and that such events somehow escaped detection.) Unexpectedly, the Atf1-11M-HA protein still activated *M26* DNA site-dependent recombination and did so to the same extent as did wild type Atf1 and Atf1-HA (**Figure 3C**). Additional control experiments revealed no recombination hotspot activity for Atf1-HA in *spc1Δ* mutants, so the HA epitope tag does not suppress the requirement for Spc1 or for Spc1-dependent phosphorylation. We conclude that the Spc1-dependent phosphorylation of Atf1 at its MAPK phospho-acceptor sites is dispensable for promoting meiotic recombination at *ade6-M26*, even though the kinase itself is essential for hotspot activity (**Figure 2**).

### Tethering of Atf1 to the chromosome bypasses the requirement for the Spc1 kinase in hotspot recombination

**Figure 4:**
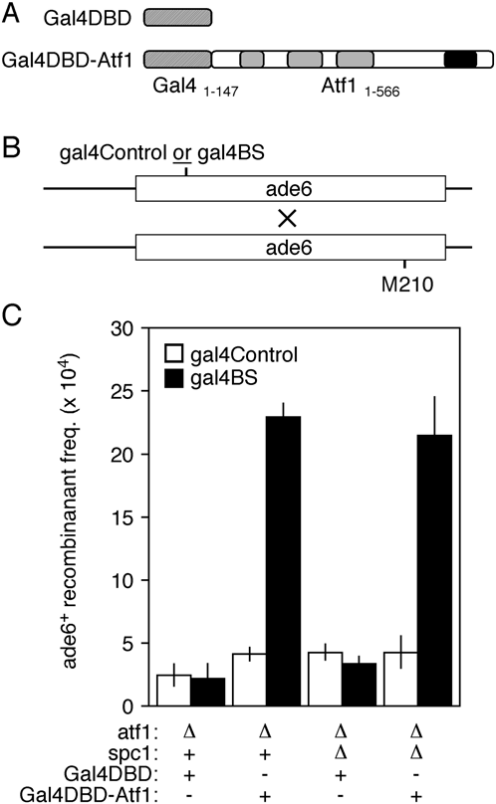
Effects of Spc1 protein kinase upon hotspot recombination after Atf1 is brought to the chromosome. (A) The 5′ and 3′ regulatory regions of the *atf1^+^* gene were used to drive expression of the indicated proteins. These contain the DNA binding domain of Gal4 protein (Gal4DBD). (B) Recombination assay. Strains harboring the indicated *ade6* alleles were crossed and the meiotic products were genotyped to determine the frequencies of *ade6^+^* recombinants. (C) Recombinant frequencies. Cells of the indicated genotypes and expressing the indicated proteins were tested for their proficiencies of recombination for *ade6* alleles with (*gal4BS*) or without (*gal4Control*) a Gal4 DNA binding site.

The Spc1 kinase can be recruited to Atf1-dependent promoters on the chromosome in an Atf1-dependent fashion, suggesting that Spc1 might gain access in *cis* to chromosomal targets [41]. Hypothetically, the recruitment of Spc1 kinase to the chromosome by Atf1 might promote recombination. This hypothesis could explain why the Spc1 kinase is essential for hotspot activity (**Figure 2**), even though canonical Spc1-dependent phosphorylation of Atf1 is not (**Figure 3**). Consequently, we developed an approach to test this hypothesis directly (**Figure 4**).

The homologous recombination-activation domain of Atf1-Pcr1 heterodimer resides exclusively within Atf1, and the Pcr1 protein contributes DNA-binding site specificity to the Atf1-Pcr1 heterodimer, thereby bringing the recombination-activation domain of Atf1 to the *M26* DNA site in the chromosome [14]. The formation of Atf1-Pcr1-*M26* protein-DNA complex *in vivo* requires Spc1 [12]. We reasoned that if we bypassed the role for Spc1 kinase in binding of Atf1 to the chromosome, then any additional, *cis*-acting requirements for Spc1 would become manifest.

We therefore constructed yeast strains with two additional alleles within *ade6* (**Figure 1C**). Each contains a premature stop codon (K87*), thereby rendering the cells auxotrophic for adenine and providing an allele for the analysis of recombination. The first allele (*ade6-gal4BS*) contains substitutions at six nearby base pairs to create a DNA binding site for the Gal4 protein of budding yeast. The second allele, *ade6-gal4Control*, also contains six additional substitutions, but does not generate a DNA binding site for Gal4 protein. This serves, first, as a negative control for the binding of Gal4. Second, since the *ade6-gal4Control* allele has the same number and type of substitutions as *ade6-gal4BS*, it also controls for any marker effects that might be created by the base-pair substitutions themselves (*e.g.*, [42], [43]). Within the various strains we expressed alternatively two different proteins that contained, respectively, a Gal4 DNA-binding domain (Gal4DBD) and a Gal4DBD coupled to full-length Atf1 protein (Gal4DBD-Atf1) (**Figure 4A**).

Strains harboring the new *ade6* alleles were crossed to a strain with the tester allele *ade6-M210* and the recombinant frequencies were determined (**Figure 4**). When we expressed the Gal4DBD alone in *atf1Δ* mutants, the recombinant frequencies for the two alleles (*ade6-gal4BS* and *ade6-gal4Control*) were indistinguishable from each other (**Figure 4**) and were similar to those for the standard negative control allele, *ade6-M375* (**Figure 2**). Thus, neither the DNA alleles nor the Gal4DBD promote recombination at *ade6*. However, the expression of Gal4DBD-Atf1 fusion protein significantly increased recombinant frequencies for its DNA binding site (*ade6-gal4BS*) relative to recombination at the matching control allele that lacks a Gal4 DNA binding site (*ade6-gal4Control*) (**Figure 4**). We conclude that when the Atf1 protein is brought to the chromosome it is sufficient to promote recombination, at least when wild-type Spc1 kinase is present.

Having established that the tethering of Gal4DBD-Atf1 to the chromosome of *atf1Δ* mutants is sufficient to promote recombination, we next examined recombination in *atf1Δ spc1Δ* cells to test whether the Spc1 kinase was still required for hotspot activity (**Figure 4**). In cells expressing the Gal4DBD protein alone the recombinant frequencies for *ade6-gal4BS* and *ade6-gal4Control* were equivalent. However, in cells expressing Gal4DBD-Atf1 the recombinant frequency at *ade6-gal4BS* was significantly higher than that at *ade6-gal4Control*. Furthermore, the frequency of recombinants when Atf1 was brought to the chromosome in cells lacking Spc1 protein was indistinguishable from that of cells expressing Spc1 protein. We conclude that the Spc1 protein kinase is not required to promote hotspot recombination after Atf1 is brought to the chromosome.

## Discussion

**Figure 5:**
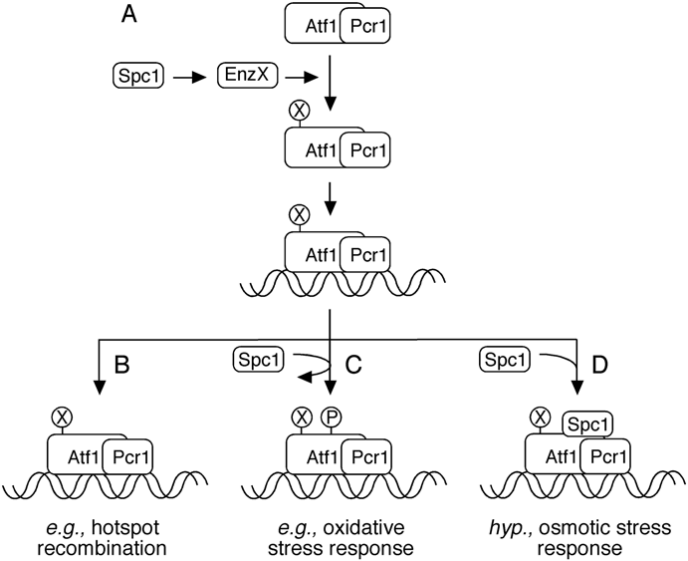
Model in which the stress-activated protein kinase Spc1 regulates multiple steps in the Atf1-mediated pathways of stress responses and hotspot recombination. (A) The Spc1 kinase regulates the DNA binding of Atf1-Pcr1 heterodimer, and hence all DNA binding-dependent functions. This regulation does not require the phosphorylation of Atf1-Pcr1 heterodimer and is likely exerted indirectly via other post-translational modifications (X). (B) Some functions of Atf1, such as promoting meiotic recombination, require no further regulation by the Spc1 kinase. (C) Some functions of Atf1, such as in the oxidative stress response, apparently require (*i.e.*, are regulated by) the phosphorylation (P) of Atf1 by the Spc1 kinase. (D) Some functions of Atf1, such as in the osmotic stress response, require hypothetically (*i.e.*, are regulated by) the binding of Spc1 to Atf1. Three Spc1-regulated, binary switches (±X, ±P, and ±bound Spc1) would provide combinatorially eight potentially distinct functional states to Atf1. According to this model, the Spc1 kinase could regulate additional, Atf1-dependent functions that are exerted independent of the binding of Atf1-Pcr1 heterodimer to the chromosome (not shown).

In this study we further defined the pathway by which the Spc1 protein kinase regulates Atf1-promoted meiotic recombination at the *cis*-acting hotspot *ade6-M26* in the chromosome. In addition, we gained new insight into regulatory mechanisms for shared (*e.g.*, DNA binding) and distinct (*e.g.*, osmoregulatory *vs.* recombinogenic) activities of the multifunctional, stress-activated protein Atf1 (**Figure 5**).

### Spc1 kinase-dependent, Atf1-phosphorylation-independent regulation of Atf1-promoted recombination

Binding of Atf1-Pcr1 heterodimer to *M26* (*CRE*-like) DNA sites within *ade6* and elsewhere in the genome promotes the initiation of meiotic recombination by Rec12 (Spo11) [11], [13], [16], [17]. The recombination-promoting domain resides in Atf1 [14] and hotspot activity strictly requires the protein kinase Spc1 (**Figure 2**), which phosphorylates Atf1 [25], [28]. Remarkably, a version of the Atf1 protein that is not detectably phosphorylated [40] supports wild-type levels of hotspot recombination under conditions where Atf1 is rate limiting (**Figure 3**). In other words, the phosphorylation of Atf1 at its canonical phospho-acceptor sites is dispensable for Atf1-promoted meiotic recombination at its chromosomal targets. We do not mean to imply that phosphorylation is unimportant. Indeed, cells expressing phosphorylation-deficient Atf1-11M protein are sensitive to oxidative stress [40]. It thus remains possible, perhaps even likely, that some Atf1-dependent functions (*e.g.*, the oxidative stress response) are regulated directly by the phosphorylation of Atf1, even though hotspot recombination is not (**Figure 5B–C**).

### Spc1 kinase regulates the DNA binding of Atf1 by an Atf1-phosphorylation-independent mechanism

Among the many hypothetical, phosphorylation-dependent regulatory mechanisms for hotspot recombination that are disproved by our findings, one warrants further discussion. This is the regulation of protein-DNA binding that is widely assumed to be required for all Atf1-dependent functions.

The binding of Atf1-Pcr1 heterodimer to *M26* (*CRE*-like) DNA sites *in vivo* and *in vitro* is seemingly constitutive and efficient binding requires the Spc1 kinase [11], [12], [30], [44]. But paradoxically, Spc1-dependent phosphorylation of Atf1 does not regulate its DNA binding affinity, as judged by biological, molecular, and biochemical criteria: First, the phosphorylation-deficient Atf1-11M protein fully activates recombination in an *M26* DNA site-specific fashion (**Figure 3**), which requires DNA binding [11], [13]. Second, phosphorylation-deficient Atf1-11M still binds to Atf1-dependent promoter regions, as gauged by chromatin immunoprecipitation [41]. Third, native Atf1-Pcr1 heterodimer purified to near homogeneity (40,000-fold) from fission yeast cells binds to the *M26* DNA site with high affinity (K_d_∼1 nM) and this high affinity DNA binding is unaltered by the treatment of purified heterodimer with each of three different protein phosphatases [11], [12]. In contrast, recombinant Atf1-Pcr1 heterodimer purified to near homogeneity from *E. coli*, which should mimic de-phosphorylated protein from fission yeast, exhibits a substantially weaker binding affinity for the *M26* DNA site (K_d_>25 nM) [13]. We suggest that a solution to the paradox might lie in the use of other, Spc1-dependent post-translational modifications to regulate the DNA binding affinity of Atf1 (**Figure 5A**). For example, acetylation regulates the DNA binding of other transcription factors such as HNF-4, NF-kappaB, and AML [45], [46], [47]. In this scenario, the Spc1 kinase would indirectly regulate Atf1 by controlling the expression or activity or specificity of yet-unidentified factors such as acetyltransferase enzymes. It remains to be seen whether Atf1 is subject to post-translational modifications other than phosphorylation and, if so, whether such modifications are regulatory.

### Spc1 kinase-independent activation of meiotic recombination after Atf1 is brought to the chromosome

A budding yeast homolog of the Spc1 kinase, Hog1, is recruited to promoter regions by transcription factor Hot1 following osmotic stress [48]. Thereupon Hog1 recruits a histone deacetylase complex and components of the basal transcription machinery such as RNA Pol II [49], [50]. The Spc1 kinase of fission yeast is similarly recruited to Atf1-dependent promoters in an Atf1-dependent fashion [41]. We therefore hypothesized that the Spc1 kinase, brought to the chromosome by Atf1, would be required to activate Atf1-promoted (hotspot) meiotic recombination. As a direct test of this hypothesis, we employed a system in which the Atf1 protein is tethered to the *ade6* locus *via* a heterologous DNA binding domain from Gal4 protein (**Figure 4**) [14]. Our rationale was that this approach would bypass the functions of Spc1 kinase in regulating the *in vivo* binding of Atf1 to the chromosome [12], in which case any additional, *cis*-acting requirements for Spc1 would become manifest.

Remarkably, when Atf1 is tethered to the chromosome by the Gal4DBD, the normally essential Spc1 kinase (**Figure 2**) becomes dispensable for hotspot meiotic recombination (**Figure 4**). So under these conditions *all* Spc1-dependent phosphorylations of Atf1 (direct or indirect, canonical or non-canonical) are dispensable for the recombination-promoting functions of Atf1. Furthermore, the Spc1 kinase neither serves as a requisite structural adaptor for Atf1-promoted recombination, nor does it regulate (directly or indirectly) any additional *cis*- or *trans*-acting factors required to activate recombination after Atf1 protein becomes bound to the chromosome. The corollary of this conclusion is that the protein kinase Spc1 apparently regulates the Atf1-dependent pathway for hotspot meiotic recombination exclusively at or before the point where Atf1 binds to the chromosome (**Figure 5A–B**). As discussed previously, the Spc1 kinase is required for the efficient binding of Atf1 to its DNA sites *in vitro* and *in vivo*, and we posit that this regulation involves Spc1-dependent post-translational modifications other than phosphorylation.

The fact that chromosome-bound Atf1 promotes recombination equally well in the presence or absence of the Spc1 kinase (**Figure 4**) demonstrates that hotspot activity per se does not require the phosphorylation of any chromatin-associated proteins by the Spc1 kinase. It also seems clear that the Spc1 kinase does not regulate general components of the basal meiotic recombination machinery, because basal recombination for various control alleles (*e.g.*, *M375* and *gal4Control*) is Spc1-independent (**Figures 2** **,** **4**). Similarly, the Spc1-independence of Atf1-promoted recombination after Atf1 is brought to the chromosome (**Figure 4**) further supports the conclusion that the Spc1 kinase does not regulate general components of the basal recombination machinery. Rather, the Spc1-Atf1 pathway seems to regulate predominantly, if not exclusively, the correct *positioning* of recombination events catalyzed by the basal recombination machinery.

The protein kinase Spc1 is not a requisite structural adaptor or *cis*-acting regulator for Atf1-promoted recombination when Atf1 is bound to the chromosome (**Figure 4**). However, these findings do not preclude the possibility that the Spc1 kinase serves as an adaptor or *cis*-acting regulator for *other* Atf1-dependent functions on the chromosome. Indeed, given the precedence for the Hog1 (Spc1) kinase of budding yeast during osmotic stress [48], [49], we think that this is likely the case. In fission yeast the osmotic stress response and the regulated expression of about 100 osmotic stress-responsive genes require both Atf1 and Spc1 [12], [25], [33], and the osmoregulatory domain of Atf1 is distinct from that which promotes homologous recombination in meiosis [14] (depicted in **Figure 3A**). It thus would not be surprising for a subset of Atf1-dependent activities (*e.g.*, osmoregulation) to require the delivery of Spc1 kinase to Atf1 binding sites on chromosomes, even though hotspot recombination does not (**Figure 5B, 5D**).

### Summary and perspectives

Chromosome-bound Atf1 protein promotes meiotic homologous recombination. The conserved, p38-family protein kinase Spc1 regulates this pathway of Atf1-promoted (hotspot) recombination at or before the point where Atf1 binds to chromosomes, and this pathway regulation is independent of the phosphorylation status of Atf1. Regulation of basal meiotic recombination is Spc1-independent. Therefore the principal function of the Spc1 kinase in meiotic recombination is to correctly position Atf1-promoted recombination at hotspots along chromosomes. For broader context, Atf1 is a multifunctional, ATF/CREB protein with a modular functional architecture [14]. Given that the phosphorylation of Atf1 is apparently required for the oxidative stress response [40] but not for hotspot meiotic recombination (**Figure 3**), we suggest that individual functional modules of Atf1 protein can be regulated independently. And finally, we propose hypothetically that some Atf1-dependent functions require the delivery of Spc1 kinase to the chromosome, even though the regulation of meiotic recombination does not (**Figure 4**). The multi-point regulation of Atf1-mediated pathways by the Spc1 kinase provides a mechanistic basis for multiple, seemingly disparate functions of Atf1 (**Figure 5**). Since the function-specific regions of Atf1 are well conserved [14], these findings may be broadly applicable to the regulation of stress responses, sexual differentiation, and meiotic recombination in other eukaryotes.

## Materials and Methods

### Strains, media, and genetic methods

The relevant genotypes of *S. pombe* strains used are presented in the figure panels and full genotypes are listed in **Table 1**. Strains were cultured in nitrogen base liquid (NBL) or on nitrogen base agar (NBA) minimal media supplemented as necessary with the requisite amino acids and bases (100 µg/ml) [51], [52]. Synthetic sporulation agar (SPA) was prepared and used as described [53]. Standard genetic methods were used for the construction of new strains and the presence of specific alleles was determined by a combination of phenotyping (*e.g.*, for selectable markers), PCR diagnostics (*e.g.*, for mating type), plus restriction mapping and DNA sequence analyses of PCR products (*e.g.*, for alleles encoding epitope tags) [12], [53], [54]. Wild-type and phosphorylation-deficient versions of the Atf1 protein with a carboxy-terminal epitope tag were expressed from the endogenous *atf1* locus, as described [40]. The construction of plasmids encoding the Atf1, Gal4DBD, and Gal4DBD-Atf1 was previously described [14]. These plasmids, which harbor a *LEU2* selectable marker from budding yeast [55], were transformed [56] into cells with the *leu1-32* mutation and were maintained by selection for leucine prototrophy.

**Table 1:** Genotypes of *S. pombe* strains used in this study.

Strain	Genotype^1^	Source
NJ 71	*h^90^ atf1-2HA6His::LEU2 ade6-M216*	[40]
NJ 72	*h^90^ atf1-11M-2HA6His::LEU2 atf1::ura4 ade6-M216*	[40]
WSP 0494	*h^−^ ade6-M210 his3-D1*	[13]
WSP 0550	*h^+^ ade6-M210 his3-D1*	[13]
WSP 0571	*h^+^ ade6-M26 his3-D1*	[13]
WSP 0578	*h^+^ ade6-M375 his3-D1*	[13]
WSP 0599	*h^−^ his3-D1*	[13]
WSP 0643	*h^−^ ade6-M210 atf1-D15::ura4F his3-D1*	[13]
WSP 0644	*h^+^ ade6-M26 atf1-D15::ura4F his3-D1*	[13]
WSP 0646	*h^+^ ade6-M375 atf1-D15::ura4F his3-D1*	[13]
WSP 1037	*h^−^ ade6-M210 spc1::ura4 his3-D1*	[12]
WSP 1040	*h^+^ ade6-M26 spc1::ura4 his3-D1*	[12]
WSP 1044	*h^+^ ade6-M375 spc1::ura4 his3-D1*	[12]
WSP 2604	*h^−^ ade6-M210 atf1-D15::ura4F his3-D1 (pSP1gal4DBD-atf1)*	[14]
WSP 2905	*h^+^ ade6-gal4BS atf1-D15::his3F his3-D1*	[14]
WSP 2907	*h^+^ ade6-gal4Control atf1-D15::his3F his3-D1*	[14]
WSP 2924	*h^+^ ade6-gal4BS atf1-D15::his3F his3-D1 (pSP1gal4DBD-atf1)*	[14]
WSP 2926	*h^+^ ade6-gal4Control atf1-D15::his3F his3-D1 (pSP1gal4DBD-atf1)*	[14]
WSP 2991	*h^+^ ade6-gal4BS atf1-D15::his3F his3-D1 (pSP1gal4DBD)*	[14]
WSP 3001	*h^+^ ade6-gal4Control atf1-D15::his3F his3-D1 (pSP1gal4DBD)*	[14]
WSP 3011	*h^−^ ade6-M210 atf1-D15::ura4F his3-D1 (pSP1gal4DBD)*	[14]
WSP 3341	*h^+^ ade6-gal4BS atf1-D15::his3F spc1::ura4 his3-D1*	This study
WSP 3343	*h^+^ ade6-gal4Control atf1-D15::his3F spc1::ura4 his3-D1*	This study
WSP 3345	*h^−^ ade6-M210 atf1-D15::his3F spc1::ura4 his3-D1*	This study
WSP 3400	*h^+^ ade6-gal4BS atf1-D15::his3F spc1::ura4 his3-D1 (pSP1gal4DBD)*	This study
WSP 3402	*h^+^ ade6-gal4BS atf1-D15::his3F spc1::ura4 his3-D1 (pSP1gal4DBD-atf1)*	This study
WSP 3404	*h^+^ ade6-gal4Control atf1-D15::his3F spc1::ura4 his3-D1 (pSP1gal4DBD)*	This study
WSP 3406	*h^+^ ade6-gal4Control atf1-D15::his3F spc1::ura4 his3-D1 (pSP1gal4DBD-atf1)*	This study
WSP 3408	*h^−^ ade6-M210 atf1-D15::his3F spc1::ura4 his3-D1 (pSP1gal4DBD)*	This study
WSP 3410	*h^−^ ade6-M210 atf1-D15::his3F spc1::ura4 his3-D1 (pSP1gal4DBD-atf1)*	This study
WSP 3478	*h^−^ atf1-11M-2HA6His:LEU2 atf1::ura4*	This study
WSP 3486	*h^−^ atf1-2HA6His:LEU2*	This study
WSP 3513	*h^+^ ade6-M26 atf1-11M-2HA6His:LEU2 atf1::ura4*	This study
WSP 3522	*h^+^ ade6-M375 atf1-11M-2HA6His:LEU2 atf1::ura4*	This study
WSP 3530	*h^−^ ade6-M210 atf1-11M-2HA6His:LEU2 atf1::ura4*	This study
WSP 3537	*h^+^ ade6-M26 atf1-2HA6His:LEU2*	This study
WSP 3544	*h^+^ ade6-M375 atf1-2HA6His:LEU2*	This study
WSP 3549	*h^−^ ade6-M210 atf1-2HA6His:LEU2*	This study
WSP 3858	*h^−^ ade6-M210 spc1::ura4 his3-D1 (pSP1-atf1)*	This study
WSP 3860	*h^−^ ade6-M210 spc1::ura4 his3-D1 (pSP1)*	This study
WSP 3861	*h^+^ ade6-M26 spc1::ura4 his3-D1 (pSP1)*	This study
WSP 3903	*h^+^ ade6-M26 spc1::ura4 his3-D1 (pSP1-atf1)*	This study
WSP 4890	*h^+^ ade6-M26 atf1-2HA6his::LEU2 spc1::ura4*	This study
WSP 4894	*h^−^ ade6-M210 atf1-2HA6his::LEU2 spc1::ura4*	This study

1 All strains were also *ura4-D18 leu1-32*.

### Analysis of meiotic recombinant frequencies

Procedures used to induce mating, meiosis, and preparation and plating of ascospores were as described [52], [57] with several minor changes to help address the fact that the *atf1Δ* and *spc1Δ* mutants are sensitive to stress conditions and have reduced mating efficiency and spore viability [12], [26]. The changes were as follows. First, cells to be mated were harvested from NBL cultures at a lower cell density (≤5×10^6^ cells per ml). Second, after cells were mated on SPA the mating mixtures were resuspended and washed with 1% glucose, rather than H_2_O. Glucose (1%) was included in all subsequent steps. Third, after treatment of mating mixtures with glusulase, the spore suspensions were treated with a lower final concentration of ethanol (15%) than previously employed (30%). Fourth, spores were plated immediately after harvesting, because the mutants have a defect in spore quiescence [12]. Fifth, all spore dilutions were made in NBL, rather than H_2_O. Meiotic recombinant frequencies were determined by plating of spores on NBA in the presence and absence of adenine to determine the total viable titer and *ade6^+^* recombinant titer, respectively. For each cross and each experimental repeat and each plating condition, we counted at least 100 colonies to determine the respective titers. The recombinant frequency is (*ade6^+^* titer/viable titer) and the data are mean±standard deviation from three or more independent experiments.
